# Improved maternal–fetal outcomes among emergency obstetric referrals following phone call communication at a teaching hospital in south western Uganda: a quasi-experimental study

**DOI:** 10.1186/s12884-022-05007-0

**Published:** 2022-09-05

**Authors:** Hamson Kanyesigye, Jerome Kabakyenga, Edgar Mulogo, Yarine Fajardo, Daniel Atwine, Noni E. MacDonald, Robert Bortolussi, Richard Migisha, Joseph Ngonzi

**Affiliations:** 1 Department of Obstetrics and Gynecology, Faculty of Medicine, Mbarara University of Science and Technology, Mbarara, Uganda; 2 Department of Community Health, Mbarara University of Science and Technology, Mbarara, Uganda; 3 Department of Clinical Research, SOAR Research Foundation, Mbarara, Uganda; 4 Faculty of Medicine & MicroResearch International, Dalhouise University, Halifax, Canada; 5 Department of Physiology, Faculty of Medicine, Mbarara University of Science and Technology, Mbarara, Uganda

**Keywords:** Phone call, Communication, Intervention, Emergency, Obstetric referral, Quasi-experimental, Maternal–fetal outcomes

## Abstract

**Background:**

Emergency obstetric referrals develop adverse maternal–fetal outcomes partly due to delays in offering appropriate care at referral hospitals especially in resource limited settings. Referral hospitals do not get prior communication of incoming referrals leading to inadequate preparedness and delays of care. Phone based innovations may bridge such communication challenges.

We investigated effect of a phone call communication prior to referral of mothers in labour as intervention to reduce preparation delays and improve maternal–fetal outcome at a referral hospital in a resource limited setting.

**Methods:**

This was a quasi-experimental study with non-equivalent control group conducted at Mbarara Regional Referral Hospital (MRRH) in South Western Uganda from September 2020 to March 2021. Adverse maternal–fetal outcomes included: early neonatal death, fresh still birth, obstructed labour, ruptured uterus, maternal sepsis, low Apgar score, admission to neonatal ICU and hysterectomy. Exposure variable for intervention group was a phone call prior maternal referral from a lower health facility. We compared distribution of clinical characteristics and adverse maternal–fetal outcomes between intervention and control groups using Chi square or Fisher’s exact test. We performed logistic regression to assess association between independent variables and adverse maternal–fetal outcomes.

**Results:**

We enrolled 177 participants: 75 in intervention group and 102 in control group. Participants had similar demographic characteristics. Three quarters (75.0%) of participants in control group delayed on admission waiting bench of MRRH compared to (40.0%) in intervention group [*p* =  < 0.001]. There were significantly more adverse maternal–fetal outcomes in control group than intervention group (obstructed labour [*p* = 0.026], low Apgar score [*p* = 0.013] and admission to neonatal high dependency unit [*p* =  < 0.001]). The phone call intervention was protective against adverse maternal–fetal outcome [aOR = 0.22; 95%CI: 0.09—0.44, *p* = 0.001].

**Conclusion:**

The phone call intervention resulted in reduced delay to patient admission at a tertiary referral hospital in a resource limited setting, and is protective against adverse maternal–fetal outcomes. Incorporating the phone call communication intervention in the routine practice of emergency obstetric referrals from lower health facilities to regional referral hospitals may reduce both maternal and fetal morbidities.

**Trial registration:**

Pan African Clinical Trial Registry PACTR20200686885039.

## Background

Emergency obstetric referrals contribute greatly to adverse maternal and fetal outcomes among women referred from lower level health centres to regional referral hospitals [1–3]. This presents a big challenge especially in resource limited settings, such as Uganda where health indicators are poor [4]. Maternal mortality rate and perinatal mortality rate for Uganda are high at 336 per 100,000 live births and 38 per 1000 pregnancies respectively [5]. The challenges of maternal referrals and their poor outcomes are largely attributed to delays in the inefficient referral systems [3, 6]. Although patient-related delays exist, studies have demonstrated that there are additional delays in providing emergency obstetric care even after the patients have arrived at the referral hospitals [7, 8].

There are challenges of communication between the lower health centres and tertiary hospitals in resource limited settings [9]. The current standard of care in Uganda would require lower health centres to give a medical referral form (HMIS FORM 32) to a patient (emergency obstetric referral) to communicate health information of that particular patient to a referral hospital which then uses the same form to give feedback to the lower health centres concerning patient management, maternal–fetal outcomes and possible areas to improve in case management [10]. The health workers at the tertiary referral hospitals do not get prior information of which patients are being referred, when they are being referred, and the referral diagnoses [11]. The lack of an alerting communication to referral hospitals leads to inadequate preparedness, delays to offering of appropriate care and consequent bad maternal and fetal outcomes [12]. Additionally, the current feedback method of using feedback section on referral letter by referral hospitals is not very feasible and in some settings the feedback rate is zero [13] This is disadvantageous because feedback would break a vicious cycle of poor obstetric referral management at lower health centres and bad obstetric outcomes at referral hospitals [14].

However, a low cost innovation may improve real time communication between tertiary referral hospitals and lower level health centres and subsequently reduce the adverse maternal and fetal outcomes among women in labour [15]. The use of phone call technology has demonstrated improvement in functioning of maternal referral systems in Africa thereby improving morbidity and mortality among women in labour [16, 17]. In Uganda there is no prereferral phone call communication from lower level health facility to the referral hospitals. However, a pilot study revealed that the lower level health centres are willing to call referral hospital before referring mothers in labour [7].

Mbarara regional referral hospital (MRRH) in South western Uganda has a high caesarean section rate of 39%. The hospital also registers more adverse maternal–fetal outcomes among intra-partum referrals compared to non-referrals [18]. This study investigated the effect of a phone call communication prior to referral of mothers in labour on maternal and fetal outcomes at a regional referral hospital. We hypothesized that the phone call intervention would reduce delays in admitting and managing the mothers referred in labour at the referral hospital resulting in improved maternal and fetal outcomes.

## Methods

### Study setting

This study was conducted on maternity unit at Mbarara Regional Referral Hospital (MRRH), a teaching hospital for Mbarara University of Science and Technology (MUST) medical school in South Western Uganda. The hospital is a 350-bed tertiary care capacity and conducts approximately 12,000 deliveries annually. The maternity unit of MRRH receives an average of 120 emergency obstetric referrals per month, with approximately 40% of referrals coming from Isingiro district alone. The majority of referrals from Isingiro district come from Kabuyanda and Rwekubo health centre IVs. Being health centre IVs; Kabuyanda and Rwekubo are headed by a general Doctor, have operating theatre but unable to provide advanced obstetric care [19], and often refers mothers for caesarean section to a regional hospital because they are unable to work on all the cases 24 h a day.

These two health centres neighbor each other and are approximately 50 km from MRRH. Patients are routinely referred without prior communication to MRRH and present to the hospital with a referral medical form; HMIS FORM 32 [10], which is a standard of care. Once they have reached MRRH, patients wait at admission bench until the health worker is ready to call them into the examination room for triage assessment and admission to maternity unit. This is because the emergencies come in unannounced and without being escorted by a health worker. As such, health workers at referral hospital may not be aware that such an emergency is waiting among other patients in the triage area.

### Study design

This study employed a quasi-experimental design with non-equivalent control group. By tossing of a coin, Rwekubo HCIV was selected to implement the intervention while Kabuyanda HCIV maintained standard of care. The referrals from Rwekubo HCIV formed the Intervention group while the ones from Kabuyanda HCIV followed standard of care which was; use of referral form only with no phone call. The referrals from Kabuyanda HCIV formed the Control group.

The intervention involved a real-time pre-referral phone call from health centre IV by health care workers (HCWs) at health centre IV to alert a contact person at MRRH, and the contact person would alert the responsible team on call at MRRH maternity ward to be prepared for management of obstetric referral. The HCV was given a preloaded phone and a specific phone number of MRRH maternity unit to call in order to facilitate prereferral phone calls. The phone was stationed and operated by the midwife on duty at HCIV and would be handed over from shift to shift. We loaded 8 US dollar monthly airtime for all the study period. The HCWs implementing the intervention did not have to be trained because they already knew how to use a phone; however, the principal investigator briefed them about the study and the need to implement the intervention. The phone call was in addition to the standard of care (referral forms). Upon discharge, the health centre implementing the intervention was also called by the research team at MRRH for feedback stating the treatment which MRRH gave to the patient and maternal–fetal outcomes so that the referral process was complete.

On the other hand, no phone call was made to the control (comparator) health centre IV (Kabuyanda HCIV) to give feedback upon patient discharge, they maintained the standard of care.

Standard of care was that once a decision to refer a mother in labour was made, the health worker at Kabuyanda HCIV would fill a referral form (HMIS Form 32) and give it to the mother. This form shows details of mother being referred including biodata, referral diagnosis, reason for referral, pre-referral treatment and the possible intervention required. The mother would present this referral letter at MRRH and no prior communication would be made from Kabuyanda HCIV to MRRH. The last section of HMIS Form 32 is supposed to be used by the health workers at MRRH to give feedback to Kabuyanda HCIV.

The rationale for use of quasi-experimental study was to account for complexity of studying maternal referral processes from specific lower health centres to regional referral hospital. Also, it was only ethical to allow the tertiary referral hospital manage the emergency obstetric referrals who participated in the study the same way other referrals from different health centres were managed. Once the referrals arrived at MRRH, they were all treated according to the hospital standard protocols of care without adjustment by the research team. The selection bias was reduced by use of a comparator arm composed of a population with similar characteristics.

### Study participants

Eligible subjects were emergency obstetric referrals from Isingiro district, referred from Kabuyanda HC IV and Rwekubo HC IV to MRRH for delivery during September 2020-March 2021. Consecutively, the referrals from Rwekubo formed the Intervention group while the control group had only patients referred from Kabuyanda HC IV. Kabuyanda HCIV and Rwekubo HCIV were considered to be similar in that they are all Government health centre IVs with similar cadre in staffing (with a medical doctor heading the team of midwives and nurses), share patient management protocols and are supervised by the same district health officials, and almost equidistant from MRRH. The residents in the area share same geographical, cultural and economic characteristics. Therefore, the referrals in both the intervention group and control group to MRRH were thought to have similar characteristics which would reduce the selection bias brought by lack of randomization of study participants.

We got informed consent from all participants (emergency maternal referrals) and we excluded all patients who did not have referral forms as we could not ascertain evidence of the health centre that had referred them.

### Sample size estimation

We used a formula of sample size calculation for comparison of proportions in experimental studies [20]. We assumed power of 80% to detect a 20% clinically important difference of maternal–fetal outcomes between the intervention and the control group, from an estimated source population of 226 women expected from Rwekubo and Kabuyanda HCIVs over the study period of six months. The 20% difference was based on a mobile phone intervention study in India which improved both antenatal registration and delivery in hospital by approximately 20% [21]. We considered the two health centres (one implementing the intervention and another for the control group) as two clusters. After adjusting the sample size for design effect resulting from clustering of observations at health centre level, with intra-cluster correlation coefficient of 0.01 based on estimates in human studies [22], we estimated a sample size of at least 106 for both intervention and control group (53 participants per arm).

### Data collection and study variables

We collected data using pretested Case Report Forms (CRFs) that captured the different study variables including demographics, obstetric factors, medical factors, health system factors, phone call before referral, delays and maternal–fetal outcomes. Trained research assistants who were midwives administered the CRFs. All study participants were followed up for a whole period from admission until discharge from the hospital during which adverse maternal–fetal outcomes were documented. The follow up of study participants was done by the Principal Investigator and the research assistants who captured the study variables prospectively. The study participants were asked questions and examined once a day while in hospital (MRRH) with the aim of identifying the maternal and fetal outcomes.

The primary outcome of interest in this study was adverse maternal–fetal outcome. Adverse maternal–fetal outcome was considered to be any of the following; early neonatal death, Fresh still birth, obstructed labour, caesarean delivery, ruptured uterus, maternal sepsis, Apgar score at 5 min below seven, admission to a neonatal ICU, disability to the mother like total abdominal hysterectomy, and organ failure. The diagnoses and other outcomes were considered as documented by the clinical team of MRRH led by Obstetrician-Gynecologist on duty.

The major exposure variable for the interventional arm was the phone call. The other independent variables included the factors causing the delays as depicted by the three delays model of Thaddeus and Maine [23]. They included (i) factors that affect individual’s utilization of hospital which included woman's social demographics (education level, financial capability, age, gravidity and parity, (ii) factors affecting accessibility (availability of transport means and transport costs, long distances) and (iii) factors relating to the quality of medical care characterized by long waiting hours (delays). Once the referral arrived at the admission waiting bench of MRRH, the research assistant would note the time and observe the patient all through the process of triage, admission and receiving care to ensure correct timing of delays. These research assistants worked in shifts and did data collection every day for 24 h. We defined delay at the admission waiting bench as a participant who took more than 30 min from arrival at admission waiting bench to getting examined and admitted by the health worker on duty at MRRH (mid wife or doctor). This is more than the international standard of 10 min for triage [24]. We used cutoff of 30 min for a delay because to be examined and admitted takes more than just triage in our setting and also we felt that an emergency patient taking more than 30 min on the admission waiting bench would clearly separate hospital system problem from individual patient’s challenges. Time from decision to delivery by cesarean section of > 30 min was considered a delay basing on previous study [25]. Presenting problems and diagnoses for each patient were documented as recorded in the patient admission records by the clinical care team headed by the Obstetrician-Gynecologist on duty.

### Statistical analysis

Data were entered into REDCap by trained Research Assistants, and exported to Stata 15 (StataCorp, Texas, USA) for analysis. We described demographic, obstetric and clinical characteristics, as well as maternal–fetal outcome of the study population. We compared the distribution of characteristics and maternal–fetal outcome between the Intervention and the control groups using Chi square or Fisher’s exact test. We performed univariable and multivariable logistic regression to assess the association between independent variables and adverse maternal–fetal outcome. We considered phone call as our major exposure variable of interest, and adjusted for other variables, including delay on admission bench, maternal age and parity as potential confounders. We reported Odds Ratios (ORs) with their corresponding 95% confidence intervals (CIs). The α-level of significance was set at 0.05.

## Results

During the study period, 177 eligible emergency referrals were received in labour at MRRH from the health centres participating in the study. Seventy-five (75) emergency referrals in the intervention group (referrals from Rwekubo HCIV) while one hundred and two (102) were in the control group (referrals from Kabuyanda HCIV).

The intervention and control groups had similar social-demographic characteristics with regard to age, education level, marital status, transport means to the referral hospital, occupation, and income status (Table 1).

**Table 1:** Socio-demographic profiles of emergency obstetric referrals to MRRH

**Characteristic**	**Overall (*N* = 177)**	**Received communication (*n* = 75)**	**No communication (*n* = 102)**	
	**n (%)**	**n (%)**	**n (%)**	***P* value**
**Age category in years**				0.685
16–24	100 (56.5)	40 (53.3)	60 (58.8)	
25–34	62 (35.0)	29(38.7)	33 (32.4)	
≥ 35	15 (8.5)	6 (8.0)	9 (8.8)	
**Gravidity**				0.956
1	63 (35.6)	27 (36.0)	36 (35.3)	
2–4	80 (45.2)	33 (44.0)	47 (46.1)	
> 4	34 (19.2)	15 (20.0)	19 (18.6)	
**Gestational age (weeks) (*n* = 131)**				0.490
28–36	10 (7.6)	3 (5.5)	7 (9.2)	
37–40	93 (71.0)	42 (76.4)	51 (67.1)	
> 40	28 (21.4)	10 (18.2)	18 (23.7)	
**Marital status**				0.826
Single	2 (1.1)	1 (1.3)	1 (1.0)	
Married	167 (94.4)	71 (94.7)	96 (94.1)	
Divorced	8 (4.5)	3 (4.0)	5 (4.9)	
**Level of education**				0.149
None	17 (9.6)	10 (13.3)	7 (6.9)	
Primary	111 (62.7)	43 (57.3)	68 (66.7)	
Secondary	33 (18.6)	16 (21.3)	17 (16.7)	
Tertiary	18 (10.2)	7 (9.3)	11 (10.8)	
**Occupation**				
None	155 (87.6)	63 (84.0)	92 (90.2)	0.217
Farmer	10 (5.7)	3 (4.0)	7 (6.9)	0.415
Others^b^	25 (14.1)	9 (12.0)	16 (15.7)	0.598
**Transport means**				0.496
Ambulance	109 (61.6)	27 (57.4)	82 (63.1)	
Other means^a^	68 (38.4)	20 (42.6)	48 (36.9)	
**Monthly income**				
< 75 k	122 (68.9)	49 (65.3)	73 (71.6)	0.376
75-150 k	19 (10.7)	6 (8.0)	13 (12.8)	0.314
> 150 k	21 (11.9)	9 (12.0)	12 (11.8)	0.962

For income, 1 k = 1,000 Ugandan shillings approximating to 0.27 USD

^a^Other transport means comprised use of motorcycle taxis (*n* = 9), private cars (*n* = 50), public taxis (*n* = 9)

^b^Other occupations comprised trader (*n* = 1), teacher (*n* = 5), Hair dressing (*n* = 7), tailor (*n* = 8), social worker (*n* = 3), midwife (*n* = 1)

Most mothers referred in labour were young (aged 16–24 years) with 53.3% in the intervention group and 58.8% in the control group, a big percentage for both study groups (84.0% in the intervention group and 90.2% in the control group) had no occupation. Likewise, the monthly income for majority of the participants (65.3% for the intervention group and 71.6% for the control group) was below 20 dollars. Most of the participants used ambulance as the means of transport (57.4% in the intervention group and 63.1% in the control group).

There was no significant difference in the admission diagnoses of the emergency referrals between the intervention and control groups (Table 2).

**Table 2:** Delays, diagnoses at admission and maternal–fetal outcomes among study participants

Characteristic	Overall (*N* = 177)	Received communication (*n* = 75)	No communication (*n* = 102)	
	n (%)	n (%)	n (%)	*P* value
**Delay at admission bench**				< 0.001
< 30 min	71 (40.1)	45 (60.0)	26 (25.5)	
30–60 min	49 (27.7)	27 (36.0)	22 (21.6)	
> 60 min	57 (32.2)	3 (4.0)	54 (52.9)	
**Delay from admission to resuscitation**				0.973
< 30 min	115 (65.0)	48 (64.0)	67 (65.7)	
30–60 min	32 (18.1)	14 (18.7)	18 (17.7)	
> 60 min	30 (17.0)	13 (17.3)	17 (16.7)	
**Decision to incision time** **(***n* ^a^** = 109)**				0.613
< 30 min	21 (19.3)	10 (22.2)	11 (17.2)	
30–60 min	36 (33.0)	16 (35.6)	20 (31.3)	
> 60 min	52 (47.7)	19 (42.2)	33 (51.6)	
**Admission diagnosis**				0.067
Malpresentation	12 (6.8)	6 (8.0)	6 (5.9)	
Malposition	17 (9.6)	3 (4.0)	14 (13.7)	
Fetal distress	5 (2.8)	2 (2.7)	3 (2.9)	
Inadequate pelvis	20 (11.3)	7 (9.3)	13 (12.8)	
Preeclampsia	4 (2.3)	1 (1.3)	3 (2.9)	
Premature labour	10 (5.7)	1 (1.3)	9 (8.8)	
Previous c/s scar	43 (24.3)	23 (30.7)	20 (19.6)	
Prolonged labour	34 (19.2)	14 (18.7)	20 (19.6)	
Prelabor rupture of membranes	13 (7.3)	6 (8.0)	7 (6.9)	
Others	19 (10.7)	12 (16.0)	7 (6.9)	
**Maternal outcomes**				
Obstructed labour	24 (13.6)	5 (6.7)	19 (18.6)	0.026
Caesarean section	109 (61.6)	45 (60.0)	64 (62.8)	0.711
Other complications ^¶^	6 (3.4)	0 (0.0)	6 (5.9)	0.074
**Fetal outcomes**				
Apgar score < 7	34 (19.2)	8 (10.7)	26 (25.5)	0.013
Fresh still birth	12 (6.8)	3 (4.0)	9 (8.8)	0.242
Early neonatal death	1 (0.6)	1 (1.3)	0 (0.0)	0.424
Admitted to neonatal unit	26 (14.7)	3 (4.0)	23 (22.6)	< 0.001
Any adverse maternal–fetal outcome				< 0.001
Yes	47 (26.5)	9 (12.0)	38 (37.2)	
No	130 (73.5)	66 (88.0)	64 (62.8)	
**Number of adverse maternal–fetal outcome(s) to each participant** **(*n*** **= 47) **^b^				0.697
One	32 (68.1)	7 (77.8)	25 (65.8)	
More than one	15 (31.9)	2 (22.2)	34.2)	

^a^ Only considered among 109 mothers that underwent caesarean section

^¶^ Other complications comprised sepsis (*n* = 3), ruptured uterus (*n* = 2), hysterectomy (*n* = 1)

Adverse maternal–fetal outcome was defined as any of the following: early neonatal death, fresh stillbirth, obstructed labour, ruptured uterus, maternal sepsis, Apgar score at 5 min < 7, admission to a neonatal unit, hysterectomy, or organ failure

^b^ Considered among those with any maternal–fetal complication (*n* = 47)

Cesarean section rates were similar in both groups. Although not statistically significant, some adverse outcomes of clinical importance were observed to have occurred only in the control group. These included ruptured uterus with two participants (02%), hysterectomy with one participant (01%), and sepsis with three participants (2.9%) (Table 2 under Other complications¶). There were no maternal deaths among the study participants during the study period however, there were fresh still births; 8.8% of the control group compared to 4% of the intervention group although there was no statistically significant difference (*p* = 0.242) (Table 2).

There were significantly more adverse maternal and fetal outcomes in the control group than the intervention group; these were obstructed labour (*p* = 0.026), low Apgar score (below 7) at five minutes after birth (*p* = 0.013) and admission to neonatal high dependency unit (*p* =  < 0.001) (Table 2).

After adjusting for cofounders, the phone call intervention was protective against adverse maternal–fetal outcome (adjusted OR = 0.22; 95%CI: 0.09—0.44, *p* = 0.001) (Table 3), while delay at admission bench, age and gravidity were not significantly associated with the adverse maternal–fetal outcomes.

**Table 3:** Multivariable analysis showing association between phone call intervention and adverse maternal–fetal outcome

Variable	%Adverse outcome	Unadjusted analysis		Adjusted ^*^ analysis	
	**n/N (%)**	**Crude OR (95%CI)**	***P* value**	**Adjusted OR (95%CI)**	***P* value**
**Phone call communication**					
No	38/102 (37.3)	Ref		Ref	
Yes	9/75 (12.0)	0.23 (0.10—0.51)	< 0.001	**0.22 (0.09—0.44)**	**0.001**
**Delay at admission bench**					
< 30 min	15/71 (21.1)	Ref		Ref	
30–60 min	11/49 (22.5)	1.08 (0.45 – 2.61)	0.863	0.91 (0.35 – 2.32)	0.836
> 60 min	21/57 (36.8)	2.18 (0.99 – 4.77)	0.052	1.01 (0.41 – 2.47)	0.990
**Age in years**					
16–24	24/100 (24.0)	Ref		Ref	
25–34	18/62 (29.0)	1.30 (0.63—2.65)	0.478	1.86 (0.72 – 4.78)	0.201
≥ 35	5/15 (33.3)	1.58 (0.49—5.09)	0.440	2.31 (0.42 – 12.7)	0.335
**Gravidity**					
1	17/63 (27.0)	Ref		Ref	
2–4	20/80 (25.0)	0.90 (0.43—1.91)	0.788	0.67 (0.28—1.68)	0.335
> 4	10/34 (29.4)	1.13 (0.45—2.84)	0.799	0.59 (0.14 – 2.89)	0.472

%: Percentage; *OR* Odds ratio, *CI* Confidence interval, *Ref* Reference range

^*^ Adjusted for maternal age, gravidity, and delays at the admission bench

## Discussion

The phone call communication intervention aimed at investigating whether alerting the regional referral hospital through a phone call before emergency obstetric referrals from a lower health facility could reduce delays in managing the referrals at the regional referral hospital and improve maternal–fetal outcomes. This study demonstrated that the phone call intervention was associated with reduced admission delays at Mbarara Regional Referral Hospital. The intervention was also protective against adverse maternal–fetal outcome. There was a significantly lower number of referrals with obstructed labour, neonates with low Apgar scores and admissions to neonatal high dependence unit in the intervention group. This implies that the iterative communication between lower level health facilities and regional referral hospital can improve maternal and fetal outcomes if incorporated in the maternal referral system.

Our study findings of reduced delays at the referral hospital are similar to that of a study conducted in a general hospital in South-West England which demonstrated that with electronic based referral innovation, the referral process was faster and that specialists started to review patients faster compared to the period of a paper based referral process [26]. Mobile phone interventions such as use of phone calls to link patients to care have the potential to improve perinatal clinical outcomes through improved access to targeted care and increased demand for quality services [27]. The reduced delays in access to care at the referral facility could be due to increased awareness and resultant preparation to receive the mother by the clinical care team.

Also the findings in our study are in agreement with other studies in resource limited settings which found that avoiding delays in the maternal referral system improves maternal and fetal outcomes [28, 29]. The delays in the maternal referral system lead to development of obstetric complications like prolonged labour, obstructed labour, fetal distress, and if not managed early may end in fetal or maternal mortality [30]. The delays are also associated with increasing severity of adverse maternal–fetal outcome [31]. In resource limited settings, being a maternal referral from a lower health centre to regional referral hospital is a known risk factor for obstructed labour [32]. The pathway of obstructed labour is explained by a patient experiencing delays in; making decision to seek health care, reaching a health centre and accessing medical care [6, 33]. Our intervention reduced delay 3 which was delay in accessing appropriate medical care once the patient had arrived in the hospital [6]. In particular, there was less delay at the waiting bench (on arrival before triage and admission) for the intervention group. This seemed to have reduced the chances of worsening obstructed labour in the intervention group with eventual better maternal–fetal outcomes. It is documented that obstructed labour causes maternal and fetal compromise leading to the adverse maternal–fetal outcomes [34] The fetus is mainly affected by accumulation of lactic acid due to hypoxia and anaerobic metabolism leading to fetal distress and the associated outcomes of low Apgar scores and still births [35, 36].

Reduction of admission delay may not be the only pathway through which the intervention improves maternal–fetal outcomes. We hypothesize that since the intervention involved giving feedback to the referring health centre, the health professionals from the HCIV implementing the intervention could have improved their practices by referring mothers earlier before they developed complications such as obstructed labour. Mobile phone interventions are system interventions; communication and feedback between health workers interact and influence positively different levels of patient care in the whole health system [21, 37]. This is supported by the fact that we found significantly higher proportion of participants with obstructed labour in the control group. Additionally, evidence from systematic review of clinical trials shows that audit and feedback is key in improving quality of professional practice more so when baseline compliance to recommended practice is low [38]. With the paper system, return of the paper referral with post referral feedback information section from the referral hospital to the referring health centre has been reported to be very low and in some instances zero rates of feedback [13]. This minimizes the opportunity for health care workers at lower level health centres to learn more directly the impact of timing of referral and prereferral management on maternal–fetal outcomes.

The fact that the phone call communication reduced the delays at the admission, and improved maternal–fetal outcomes means that it is an important component to improve maternal referral system. Indeed in another study in a developed country, it was found to be true that continuous interactive electronic communication between lower level health units and specialised care health centres improved number of appropriate referrals and quality of information on referral documents [39]. Compared to other mobile phone interventions which involved giving phones to patients, this innovation may be cheaper and feasible based on the fact that in this intervention only one phone is given to a health centre to call regional referral hospital prior emergency obstetric referrals. In Uganda, the Ministry of Health under decentralization program gives to all health centres primary health care funds for routine operational costs, and allows the local authorities to budget according to the needs of health centres [40]. Based on our findings, health centres should budget for such communication costs in their routine operational expenditure to ensure the intervention’s sustainability.

### Limitations

Being a quasi-experimental study design, the study may have inherent selection bias. This was however minimised by selecting a control group which had participants similar to those in the intervention group as shown by the baseline characteristics of study participants. Secondly the findings may not be generalizable to different settings since it was not a multicentre study. Finally, the follow up was only in hospital, therefore we could not study factors that could have occurred differently at the two health centre IVs and in transit to the regional referral hospital before admission. Also, the long-term outcomes after discharge from MRRH were not studied, however the targeted outcomes of importance were all captured at birth and within the follow up period during admission.

## Conclusion

The simple low-cost phone call communication prior and after maternal referral between a lower level health centre and regional referral hospital was associated with less adverse maternal–fetal outcomes among emergency obstetric referrals. Therefore, incorporating the phone call communication intervention in the routine practice of obstetric referrals from lower health centres to regional referral hospitals may reduce both maternal and fetal morbidities especially in resource limited setting like Uganda.

We recommend a follow-up multicentre study to evaluate the effect of continuous communication interaction between the lower level health centres and the regional referral hospitals. This may help to generate more generalizable findings and understanding of the possible positive change in the practices of health workers at both the referring and referral health facilities, and different pathways through which the intervention improves maternal and fetal outcomes.

## Data Availability

The datasets generated and analysed for this study are available from the corresponding author, upon request.

## Abbreviations

aOR: Adjusted Odds Ratio
CIs: Confidence Intervals
HCIV: Health Centre IV
HMIS: Health Management Information System
HCWs: Health Care Workers
ICU: Intensive Care Unit
MRRH: Mbarara Regional Referral Hospital
MUST REC: Mbarara University of Science and Technology Research Ethical Committee
OR: Odds Ratios
Ref: Reference Range
UNCST: Uganda National council for Science and Technology
